# Effect of *Lactobacillus Plantarum* Supplementation on Production Performance and Fecal Microbial Composition in Laying Hens

**DOI:** 10.1515/biol-2019-0009

**Published:** 2019-03-20

**Authors:** Hongxing Qiao, Hongtao Shi, Liheng Zhang, Yuzhen Song, Xiaojing Zhang, Chuanzhou Bian

**Affiliations:** 1 Longzihu North road NO.6 Zhengzhou city, Henan, Zhengzhou P.R.China; 2 College of Veterinary Medicine, Henan University of Animal Husbandry and Economy, Zhengzhou, Henan, Zhengzhou P.R.China; 3 Henan Microbiological Transformation Engineering Laboratory, Zhengzhou, Henan, Zhengzhou P.R.China; 4 Henan Probiotics Bio-transformation Engineering Technology Research Center, Zhengzhou, Henan, Zhengzhou P.R.China; 5 Key Laboratory of Probiotics Fermentation Traditional Chinese Medicine of Zhengzhou city, Zhengzhou, Henan, Zhengzhou P.R.China

**Keywords:** additive, fecal microbiota, *Lactobacillus plantarum*, laying hens, performance

## Abstract

The present study was performed to investigate the effects of dietary supplementation with *Lactobacillus plantarum* (CGMCC1.557) on egg production and fecal microbiota composition in laying hens. Sixty Hy-Line Brown laying hens (18 weeks old) were randomly divided into two groups. The control group was fed a basal diet only, and the test group was fed basal diet supplemented with a final concentration of 1.0 × 10^9^ CFU/mL during the 10-week experimental period. Egg production and fecal microbiota composition were both assessed in 28-week-old hens using high-throughput sequencing technology. The results showed that, compared with the control group, the test group exhibited increased laying and feed intake rates (*p* < 0.05). At the genus level, *Lactobacillus* was more abundant in the test group compared with the control group (*p* < 0.05). Conversely, *Romboutsia* was more abundant in the control group compared with the test group (*p* < 0.05). This study provides us with an insight into the potential use of *L. plantarum* as a food supplement in the laying hen industry. the study also provides us with a better understanding of the interplay between *L. plantarum* and the fecal microbiota of laying hens.

## 1 Introduction

During the past few decades, antibiotics have been widely used at subtherapeutic doses to improve growth rates and performance in the poultry industry. However, antibiotics can result in side effects including bacterial resistance; these side effects often have detrimental consequences for human health [1]. In response to this apparent threat, the European Commission (EC) decided to ban the use of antibiotics as growth promoters in livestock feed (EC Regulation NO. 1831/2003) [2]. Consequently, the poultry sector needs to address the consequences of this ban by seeking alternative strategies to promote animal performance [3]. One environmentally friendly approach to growth promotion involves the use of probiotics, which have been used in the poultry industry for decades [4, 5].

Probiotics are defined as living microorganisms that are beneficial to the host [6]. Probiotics have been shown to improve poultry performance, modulate intestinal microbiota and reduce disease risk in the poultry industry [7, 8]. Lactic acid bacteria (LAB) are the most widely used bacterial order in relation to probiotic administration. LAB include the genera *Lactobacillus*, *Lactococcus*, *Enterococcus*, and *Streptococcus* [9]. Substantial evidence indicates that LAB are beneficial to both humans and animals [10, 11]. These beneficial effects are underpinned by the capacity of LAB to prevent colonization by pathogenic organisms, enhance the natural flora, and optimize nutrient absorption from feed [12]. However, LAB species elicit differential effects due to variations pertaining to dose, administration route, age of the animals, the appetites of the animals, the quality of farm management, farm hygiene and biosecurity [13, 14].

*Lactobacillus plantarum* is generally regarded as a safe species and has been used for a long time in both animal and human food [15, 16]. Over recent decades, attention has been focused on the deployment of *L. plantarum* in commercial poultry activities. *L. plantarum* P-8 has the potential to improve metabolic activity and nutrient utilization, while also modulating the intestinal microbiota of broiler chickens [17]. *L. plantarum* BS22 has been shown to promote immune function, production performance and intestinal microbiota homeostasis in broilers [18]. Moreover, metabolite combinations generated from *L. plantarum* RI11, RG14, and RG11 strains (COM456) have been shown to improve egg production, fecal LAB population abundance, and small intestinal villus height, while also reducing fecal pH, *Enterobacteriaceae* abundance, and plasma and yolk cholesterol concentrations [19, 20].

In recent years, next-generation sequencing technology has been used to determine the composition, establishment and function of gut microbiota in chickens [21, 22]. However, only a limited number of studies pertaining to the effects of *L. plantarum* on the microbiota of laying hens have been published. In the current study, we hypothesized that supplementing feed with *L. plantarum* would improve production performance, while also modulating the host fecal microbiome of laying hens. The study also provides us with some interesting insights into the use of *L. plantarum* in laying hens.

## 2 Materials and methods

### 2.1 Experimental animals

Hy-Line Brown layers obtained from Chunya Poultry Company Limited (Xuchang, China) were allocated into cages. A total of 60 Hy-Line layers (aged 18 weeks; mean body weight 1.50 kg) were randomly assigned into 2 dietary treatment groups. Each dietary group was composed of 5 identical sub-groups containing 6 birds each. The 2 dietary groups were fed either the control diet (control group) or an *L. plantarum* (CGMCC 1.557)-supplemented diet (test group). The strain of *L. plantarum* used in this current study was isolated from silage and deposited in the China General Microbiological Culture Collection Center (CGMCC, Beijing, China). This strain was characterized by acid and bile tolerance, adhesion capacity, antibacterial activity and immunomodulatory activity [23-24]. The *L. plantarum* strains were cultured using de Man, Rogosa, and Sharpe (MRS) medium at 37°C for 18 h under anaerobic conditions. The cultured *L. plantarum* cells were subsequently centrifuged at 2000 g for 15 min at 4°C and washed three times with 0.5% NaCl solution. The *L. plantarum* was resuspended to 1.0 × 10^9^ colony-forming units (CFU)/mL using 0.5% NaCl solution. Finally, the *L. plantarum* re-suspension was mixed at a level of 1 g/kg (0.1%, m/m) each day during the 10-week experimental period to ensure that the bacterial cells in the feed were viable. This level of supplementation has been shown to have positive effects on production performance, excreta microflora, ammonia emission and nutrient utilization [25]. The hens were provided with feed and water *ad libitum* during the experimental period. No antibiotics were used throughout the trial period. The temperature was maintained at 25–27°C, with 16 h of light per day. The basal diet (Table 1) used in the present study was formulated according to the NRC (1994) recommendations, which have been shown to be suitable for laying hens. All animal experiments were conducted according to the Guidelines for the Care and Use of Experimental Animals established and approved by the Laboratory Animal Management Committee of Henan University of Animal Husbandry and Economy (HNMY1618).

**Table 1:** Composition of the basic diet

Ingredient	Amount (percentage of the dried weight)
Corn	60.51
Soybean meal	24.00
Wheat	2.80
Crude palm oil	1.25
L-lysine	0.06
DL-methionine	0.15
MDCP	2.40
Limestone	7.30
Common salt	0.50
Vitamin premix^†^	0.07
Mineral premix^‡^	0.06
Choline chloride	0.90
Total Calculated values	100
ME (cal/kg)	2805.32
Crude protein,%	16.00
Calcium,%	3.70
Aval. Phophprus,%	0.45

† Vitamin premix provided (per kilogram of diet): retinol 3.00 mg; cholecalciferol 0.06 mg; ɑ-tocopherol 15.00 mg; thiamine 1.20 mg; riboflavin 4.00 mg; pantothenic acid 8.00 mg; pyridoxine 2.00 mg; niacin 30.00 mg; cobalamin 0.02 mg; folic acid 0.50 mg; biotin 0.03 mg; menadione 3.00 mg. ^‡^Mineral premix provided (per kilogram of diet): manganese 100.0 mg; copper 8.0 mg; iodine 0.8 mg; cobalt 0.25 mg; selenium 0.3 mg; zinc 80.0 mg; iron 40.0 mg.

#### Ethical approval

The research related to animals use has been compl ied with all the relevant national regulations and institutional policies for the care and use of animal.

### 2.2 Egg production performance

Egg production, egg weight and the number of cracked eggs were recorded daily. Feed consumption was recorded weekly per replicate sub-group. Laying and feed (feed conversion ratio; FCR) efficiencies were also calculated.

### 2.3 Fecal collection and DNA extraction

Thirty fresh fecal samples (15 each from the control group and test group, 3 samples per sub-group) were randomly collected at 28 wk. The fecal samples were stored immediately at −20°C prior to DNA extraction. Two hundred milligrams of feces from each bird were utilized for DNA isolation using a DNA isolation kit (Tiangen Biotech Corporation, Beijing, China) following the manufacturer’s instructions. The quality of the extracted DNA was assessed by 0.8% agarose gel electrophoresis and spectrophotometry (optical density at 260/280 nm). All extracted DNA samples were stored at −20°C prior to further analysis.

### 2.4 Library preparation and Illumina MiSeq sequencing

NGS library preparations and Illumina MiSeq sequencing were conducted at GENEWIZ, Inc. (Suzhou, China). For the library preparation, the V3 and V4 regions of the 16S rRNA gene were amplified using a 10-ng DNA aliquot isolated from each fecal sample. The V3 and V4 regions were amplified by polymerase chain reaction (PCR) using a forward primer (5′–CCTACGGRRBGCASCAGKVRVGAAT–3′) and a reverse primer (5′–GGACTACNYVGGGTWTCTAATCC–3′). The first-round PCR products were used as templates for a second round of amplicon enrichment by PCR (94°C for 3 min, followed by 24 cycles at 94°C for 5 s, 57°C for 90 s, 72°C for 10 s and a final extension at 72°C for 5 min). At the same time, indexed adapters were added to the ends of the 16S rDNA amplicons to generate indexed libraries that were ready for downstream NGS on the MiSeq platform (Illumina, San Diego, CA, USA). DNA libraries were validated by Agilent 2100 Bioanalyzer (Agilent Technologies, Palo Alto, CA, USA) and quantified using a Qubit 2.0 Fluorometer. Next, the DNA libraries were multiplexed and loaded on an Illumina MiSeq instrument according to the manufacturer’s instructions (Illunmina, San Diego, CA, USA). Sequencing was performed using a 2 × 300/250 paired-end (PE) configuration; image analysis and base calling were performed using MiSeq Control Software (MCS) embedded in the MiSeq instrument. The sequences generated in this study have been deposited in the National Center for Biotechnology Information sequence read archive (https://www.ncbi.nlm.nih.gov/biosample) under the accession number SRA: SRS2480324.

### 2.5 Statistical analysis

The QIIME data analysis package was used for the 16S rRNA data analysis. The forward and reverse reads were joined, assigned to samples based on barcodes and truncated by cutting off the barcode and primer sequences. Quality filtering of the joined sequences was performed, and sequences that did not fulfill the following criteria were discarded: sequence length < 200 bp, no ambiguous bases, and a mean quality score ≥ 20. Next, the sequences were compared with sequences from a reference database (the Ribosomal Database Project [RDP] Gold database) using the UCHIME algorithm to detect chimeric sequences; the chimeric sequences were subsequently removed.

The quality filtered-sequences were utilized in the final analysis. Sequences were grouped into Operational Taxonomic Units (OTUs) using the clustering program VSEARCH (1.9.6) against the SILVA 119 database that was pre-clustered at a 97% sequence identity. The RDP classifier was used to assign a taxonomic category to all OTUs at a confidence threshold of 0.8. The RDP classifier uses the SILVA 119 database, which predicts taxonomic categories at the species level. Sequences were rarefied prior to calculating alpha and beta diversity indices. Alpha diversity indices were calculated in QIIME from rarefied samples, using the Shannon index for diversity and the Chao1 index for richness. Beta diversity was calculated using a PCoA analysis. A heatmap was clustered using R (2.15.3) analysis. Differences between the 2 groups were compared using STAMP (2.1.3) analysis. All results were analyzed by AVOVA using the general linear models (GLM) procedure (SAS Inst. Inc., Cary, NC) and expressed as mean ± standard errors. *p* < 0.05 was considered statistically significant.

## 3 Results

### 3.1 Egg production performance

The egg production performance results are presented in Table 2. There were significant differences in the laying rate and feed intake between control and test groups (*p* < 0.05). However, egg weight, feed conversion ratios and the number of cracked eggs between the groups were not different between groups (*p* > 0.05).

**Table 2:** Effect of *L. plantarum* on the performance of laying hens at 28 weeks of age*

Performance variable	Test group	Control group	SEM	*P*-value
Laying rate (%)	93.51^a^	90.97^b^	0.31	0.04
Egg weight (g)	55.27	55.12	0.89	0.91
Egg mass(g/hen/d)	48.89	48.69	0.72	0.86
Feed intake (g/hen/d)	108.27^a^	106.30^b^	1.54	0.03
Feed conversion ratio (g/g egg)	2.09	2.12	0.37	0.59
Cracked-egg rate (%)	0.09	0.12	0.02	0.54

a, b Means in the same row not sharing a common superscript differ significantly at *P* < 0.05. SEM = Standard error of means.

### 3.2 Data acquisition and analysis of the groups

**Figure 1:**
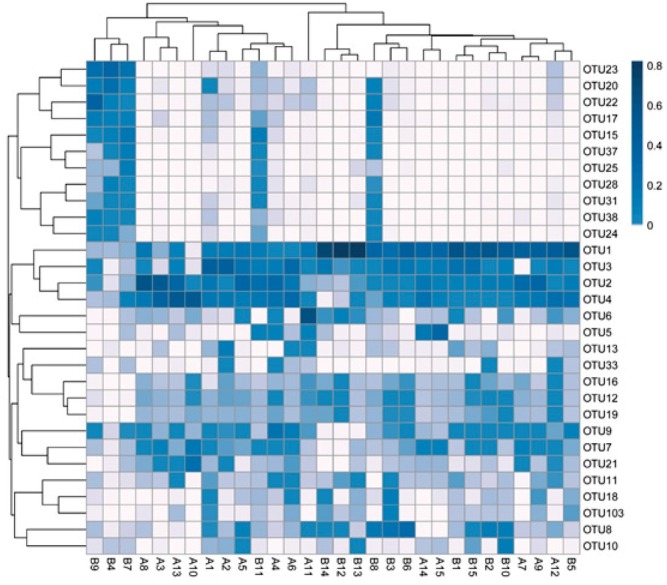
Cluster analysis of the 30 most abundant OTUs in the two groups. A: test group; B: control group.

In this study, a 16S rRNA gene sequence analysis of 30 fecal samples generated 3,505,668 high-quality sequences, with an average of 116,855 sequences per sample. The sequences were clustered into 205 OTUs using a 97% similarity cut-off. A clustering analysis of 30 OTUs with the highest abundances revealed both similarities and differences between the samples (Fig. 1). For example, similar abundances of OTU1, OTU2, OTU3 and OTU4 were

observed across nearly 30 samples, and these OTUs belong to one cluster. There were some similarities in the OTUs (OTU31, OTU28, OTU25, OTU37, OTU15) among samples B8, B4, B7, B9 and B11, while other samples showed similarities in abundances as well. Other OTUs had some dissimilarity among the rest of the samples. Using an abundance-based coverage estimator (ACE) along with Chao1, Simpson and Shannon indices, we observed the species richness between the two groups (Table 3). The Shannon index for the control group was significantly higher (*p* < 0.05) than for the test group, while there were no significant differences in the ACE, Chao1 index and Simpson index between the two groups. These results indicate that species diversity was more abundant in the test group compared with the control group.

**Table 3:** Diversity estimations for the 16S rRNA gene libraries of the 30 samples from the 16S rRNA sequences

Group	Chao1	ACE	Simpson	Shannon
Control (n = 15)	113.66 ± 33.75	117.01 ± 34.74	0.72 ± 0.08	2.47 ± 0.41^a^
Test (n = 15)	119.37 ± 38.13	121.91 ± 36.03	0.72 ± 0.20	3.10 ± 1.43^b^

a,b Different superscript letters in the same column indicate significant differences.

### 3.3 Microbial beta diversity analysis

**Figure 2:**
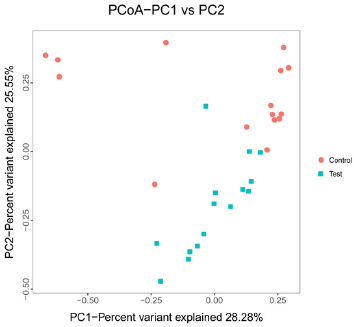
PCoA plot. Three clusters were observed for the 30 samples from the 2 groups.

In a beta-diversity analysis, variations in the composition of the microbial communities for the 30 samples were presented using principal coordinates analysis (PCoA) plots, with PC1 accounting for 28.28% of the total variation and PC2 accounting for 25.55% (Fig. 2). Thus, there were 2 main clusters between the test group and the control group; however, 3 laying hens in the control group were classified as outliers. This result suggested that the microbial population structures between the two groups were slightly different.

### 3.4 Bacterial community composition

**Figure 3:**
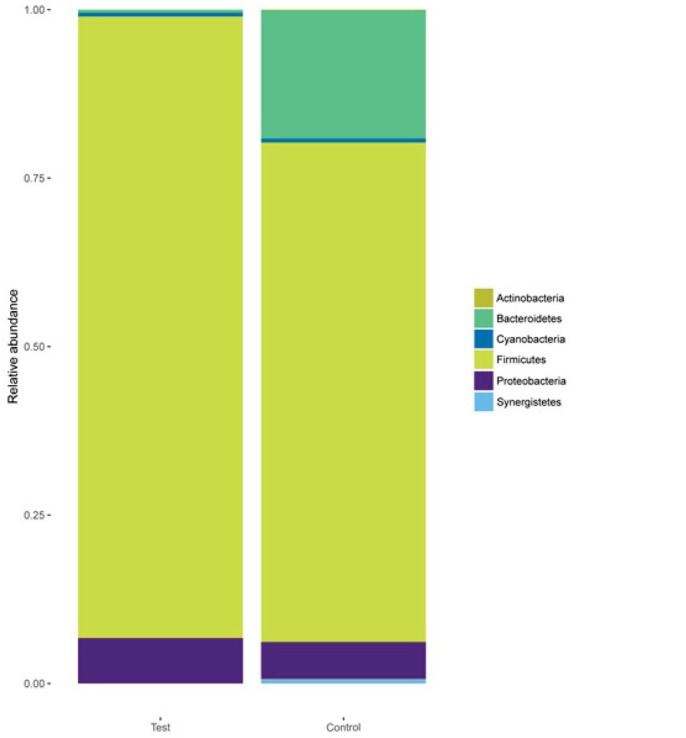
Phylum-level analyses of the 30 samples. Comparison of the relative abundances of the main bacterial phyla in the 2 groups.

At the phylum level, a total of 6 main phyla were identified in the 2 groups as shown in Fig. 3. Most of the sequences in the test group belonged to *Firmicutes* (92.24%), *Bacteroidetes* (0.43%), and *Proteobacteria* (6.69%), while *Firmicutes* (74.11%), *Bacteroidetes* (19.04%), and *Proteobacteria* (5.44%) were also abundant in the control group. The phylum-level microbiota analysis demonstrated that *Firmicutes* were the predominant bacteria in the fecal samples from laying hens. There was a significant difference between the 2 groups at the phylum level, mainly in the *Bacteroidetes* (*p* < 0.05).

**Figure 4:**
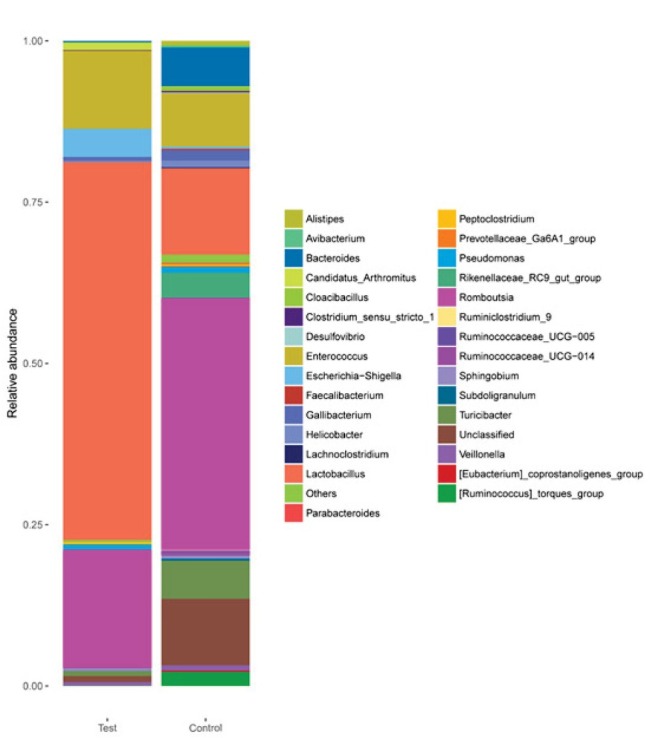
Genus-level analyses of the 30 samples. Comparison of the relative abundances of the main bacterial genera in the 2 groups.

As shown in Fig. 4, at the genus level, an analysis of the most abundant taxa revealed that *Lactobacillus* (58.55%), *Romboutsia* (18.29%), and *Enterococcus* (12.09%) were abundant in the test group, while *Lactobacillus* (13.39%), *Romboutsia* (39.02%), and *Enterococcus* (8.26%) were also abundant in the control group. The differences in the abundances of *Romboutsia* and *Lactobacillus* between the two groups were significant (*p* < 0.05). In addition, the test group contained higher *Escherichia-Shigella*, while in the control group, *Turicibater* and *Unclassed* were higher.

### 3.5 Community composition heat-map combined with a cluster analysis

**Figure 5:**
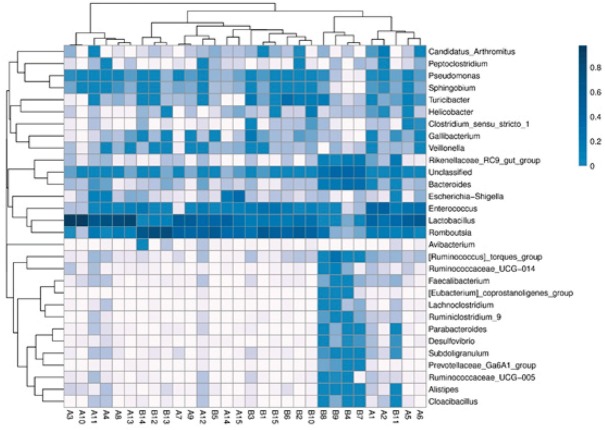
Heat-map analysis of the 30 samples. A: test group; B: control group Heat-map showing the abundances of the top 30 genera was clustered and plotted using R software. Blue represents genera with higher abundances in the corresponding sample, and white represents genera with lower abundances.

As shown in Fig. 5, the top 30 genera in terms of abundance were clustered and plotted using R software. The heat-map reveals that the abundances of *Lactobacillus*, *Romboutsia*, and *Enterococcus* were the most similar among the 30 samples. However, samples B4, B7, B8, B9 and B11 displayed the greatest diversity in relation to genus composition when compared with the other samples analyzed.

Except for the three samples B4, B7 and B9, the abundance of *Pseudomonas*, *Sphingobium* and *Turicibacter* genera was relatively high. Compared to other samples, *Escherichia-Shigella* was higher in abundance among five samples of B11, A15, A14, A4 and A11.

### 3.6 STAMP analysis

**Figure 6:**
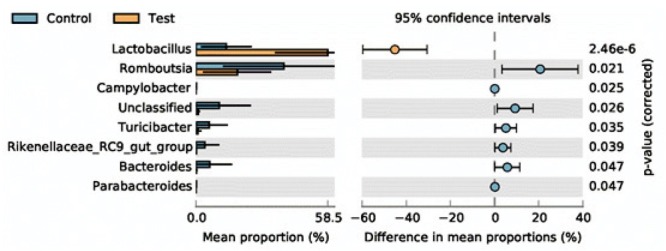
STAMP analysis of the test and control groups. *Lactobacillus* was more abundant in the test group compared with the control group.

At the genus level, we compared the differences between the 2 groups using STAMP software and Welch’s t-test. As shown in Fig. 6, the abundance of *Lactobacillus* was significantly (*p* < 0.05) higher in the test group compared with the control group. However, the abundance of *Romboutsia* was significantly (*p* < 0.05) higher in the control group compared with the test group.

## 4 Discussion

Many studies have investigated the effects of dietary supplementation with *L. plantarum* on the performance of laying hens; however, only a limited amount of research

has been performed using 16S rRNA sequencing technology to investigate fecal microbiota composition in laying hens. In this study, we observed that the fecal microbiota composition of laying hens fed a diet supplemented with *L. plantarum* was different from the control group. The results of this study support our hypothesis that feed with *L. plantarum* would improve production performance, while also modulating the host fecal microbiome of laying hens.

In this study, we observed that diets supplemented with *L. plantarum* improved feed intake and laying rate. Previous studies reported that supplementation with *Lactobacillus* improved feed efficiency in hens [26]. Additional studies reported that supplementation with octacosanol or *E. faecium* also improved egg production [27, 25], while diets supplemented with *Lactobacillus johnsonii BS15* promoted growth performance in broilers [28]. In contrast, Mahdavi et al. reported that egg production was not affected by the inclusion of LAB in the diet [29]. The associated differences resulting from supplementation with probiotics were dependent upon a number of factors including the different bacterial strains present, the ages of the animals, the effectiveness of associated farm-management practices, the method of administration used, the appetites of the animals, and the hygiene conditions of the farm [30].

At the phylum level, *Firmicutes*, *Bacteroidetes*, and *Proteobacteria* were the most common phyla in the fecal samples from the laying hens (Fig. 3). In the control group, the microbiota of laying hens was dominated by *Firmicutes*

(>50%); This result is consistent with previously published findings [31,22]. However, our results differ from results published following a study on broiler chickens published by Singh [32], where it was shown that *Proteobacteria* was the most dominant phylum. In this current study, we also observed that, upon supplementation with *L. plantarum*, the abundance of *Firmicutes* was higher in the test group compared with the control group. Moreover, the addition of *L. plantarum* increased the abundance of *Firmicutes*, but reduced the abundance of *Bacteroidetes*, which was conducive to nutrient digestion. In a separate study performed by our group, we observed that *Firmicutes* (71.39%) were abundant in young laying hens [33]. Combining the results from the two studies, we found that the abundance of *Firmicutes* was the same in young laying hens and older laying hens. However, in older hens, the abundance of *Firmicutes* decreased gradually to 58.8% at 66 weeks [31]. These results revealed that *L. plantarum* can affect fecal microbiota composition in laying hens. The reason for the latter result might be explained by the fact that *L. plantarum* can improve intestinal development and digestive ability.

As shown in Fig. 4, an analysis of the most abundant taxa revealed that at the genus level, *Lactobacillus* (58.55%), *Romboutsia* (18.29%), and *Enterococcus* (12.09%) were abundant in the test group, while *Lactobacillus* (13.39%), *Romboutsia* (39.02%), and *Enterococcus* (8.26%) were also abundant in the control group. The differences in the abundances of *Lactobacillus* and *Romboutsia* were significant (*p* < 0.05). In this study, we detected genus *Romboutsia* in fecal samples from both older and younger laying hens[33]. Analysis of the two experiments with the laying hens from the same hen farm indicated that *Romboutsia genus* might be related to the environment of the hen farm. *Romboutsia genus* has been previously observed only in human gut [34], lake sediment [35] and rat gastro-intestinal tract [36]. In our experiment, feed supplemented with *L. plantarum* promoted *Lactobacillus* abundance while inhibiting *Rombousia*. The difference in abundance of the two genera is most likely underpinned

by competitive exclusion. We speculate that the reason for this competitive exclusion may due to the reduction of pH at the intestinal (duodenum, ileum, cecum) level or the colonization of the border or intestinal villi with *L. plantarum*. Another reason may be the effect of a quorum-sensing mechanism of some strains of *L. plantarum* spp. against other bacteria, producing protein bacteriocins [37].

The heat-map and STAMP analyses revealed that *Lactobacillus* was more abundant in the test group compared with the control group. *Lactobacillus* has long been known to be beneficial in both humans and animals, and a previous study demonstrated that this genus promotes a healthy gut in animals by producing several short-chain fatty acids [38]. In this current study, *Lactobacillus* was the dominant genus, and we speculate that when used as a feed supplement, *L. plantarum* can modulate fecal microbial composition and promote nutrient digestion and absorption. In addition, *Escherichia-Shigella*, which can be associated with diarrhea, was found in five samples (B11, A15, A14, A4 and A11). However, one of the limitations of this study is the use of fecal samples instead of intestinal contents. The composition of the intestinal microbiota may better reflect the animal’s digestive function. However, taking fecal samples is more conducive to animal welfare protection. Moreover, it is well known that fecal microbial diversity and composition affect the development of the immune system in poultry [39]. This suggests that we can promote the health of livestock, and more specifically poultry, by modulating the fecal microbiome. Moreover, supplementation of diets with probiotics might prove to be an effective alternative to antibiotics [18].

## 5 Conclusion

Our data suggest that administration of an *L. plantarum* strain can increase the laying rate and feed intake of laying hens. The current study also suggests that administration of *L. plantarum* plays a vital role in the modulation of fecal microbiota. The study also provides us with a better understanding of the interplay between *L. plantarum* and the fecal microbiota of laying hens. Future investigations should be performed to help us understand the mechanism in hens fed with *L. plantarum*.
